# Present status and future directions: The restoration of root filled teeth

**DOI:** 10.1111/iej.13796

**Published:** 2022-07-19

**Authors:** Francesco Mannocci, Kerstin Bitter, Salvatore Sauro, Paolo Ferrari, Rupert Austin, Bhavin Bhuva

**Affiliations:** ^1^ Department of Endodontics Faculty of Dentistry, Oral and Craniofacial Sciences King's College London London UK; ^2^ Department of Operative and Preventive Dentistry Charité ‐ University Medicine Berlin Berlin Germany; ^3^ Departamento de Odontología, Facultad de Ciencias de la Salud Universidad CEU‐Cardenal Herrera Valencia Alfara del Patriarca Spain; ^4^ Department of Operative Dentistry University of Parma Parma Italy; ^5^ Department of Prosthodontics Faculty of Dentistry, Oral and Craniofacial Sciences King's College London London UK

**Keywords:** crown, dentine bonding, endocrowns, onlay, post, root filled teeth

## Abstract

This narrative review will focus on a number of contemporary considerations relating to the restoration of root filled teeth and future directions for research. Clinicians are now more than ever, aware of the interdependence of the endodontic and restorative aspects of managing root filled teeth, and how these aspects of treatment are fundamental to obtaining the best long‐term survival. To obtain the optimal outcomes for patients, clinicians carrying out endodontic treatment should have a vested interest in the restorative phase of the treatment process, as well as an appreciation for the structural and biomechanical effects of endodontic‐restorative procedures on restoration and tooth longevity. Furthermore, the currently available research, largely lacks appreciation of occlusal factors in the longevity of root filled teeth, despite surrogate outcomes demonstrating the considerable influence this variable has. Controversies regarding the clinical relevance of minimally invasive endodontic and restorative concepts are largely unanswered with respect to clinical data, and it is therefore, all too easy to dismiss these ideas due to the lack of scientific evidence. However, conceptually, minimally invasive endodontic‐restorative philosophies appear to be valid, and therefore, in the pursuit of improved clinical outcomes, it is important that the efficacies of these treatment protocols are determined. Alongside an increased awareness of the preservation of tooth structure, developments in adhesive bonding, ceramic materials and the inevitable integration of digital dentistry, there is also a need to evaluate the efficacy of new treatment philosophies and techniques with well‐designed prospective clinical studies.

## INTRODUCTION

Root filled teeth may fail due to either biological or structural reasons. Causes of failure include persistent or recurrent endodontic disease, unrestorable caries, restorative failure, irretrievable cusp or crown fracture, vertical root fracture or periodontal disease. Whilst endodontic research is replete with clinical studies on the success rate of root canal treatment, it is acknowledged that structural failure is the most common reason for the extraction of root filled teeth (Al‐Nuaimi et al., 2020; Nagasiri & Chitmongkolsuk, 2005). As a result, there has been increasing interest in the structural and biomechanical effects of root canal treatment and subsequent post‐endodontic restorative procedures on restoration and tooth survival.

### Present status for the restoration of root filled teeth

The endodontic‐restorative interface is currently, and importantly, a ‘hot’ topic within the endodontic community, whilst philosophies and techniques that facilitate dentine preservation are very much in ‘vogue’. The frequency of publications in relation to minimally invasive endodontic‐restorative techniques over recent years highlights the belief that residual tooth structure is a key determinant in tooth survival, whilst the results of such *in vitro* studies demonstrate the challenges of reaching tangible conclusions from the inconsistent results obtained (Plotino et al., 2017; Silva, Cabral, et al., 2021a; Silva, Versiani, et al., 2021b). However, it is certainly noteworthy to witness the level of interest in minimally invasive endodontic‐restorative concepts, despite the apparent lack of clinical data to validate these techniques (Silva et al., 2022).

Therefore, research has focused on the impact of ‘minimally invasive access cavity’ preparations on the fracture resistance of root filled teeth, as a surrogate measure for tooth survival (Marinescu et al., 2020; Saberi et al., 2020; Santosh et al., 2021). Proponents of minimally invasive endodontic techniques cite that the dentine removal, primarily in the peri‐cervical region, associated with ‘traditional’ access cavity and root canal preparation procedures may predispose the residual tooth structure to the crown and/or root fracture (Clark & Khademi, 2010).

A criticism of both historical and contemporary research is that the endodontic and restorative procedures are often considered as separate entities, rather than collectively. Based on the strength of arguments for minimally invasive ‘intra‐coronal’ endodontic procedures and the scientific evidence demonstrating the importance of ‘extra‐coronal’ tooth structure of adequate quantity and quality, it seems logical that these should be considered together for root filled teeth to have optimal outcomes. However, there remains great conjecture as to the ‘thresholds’ of dentine removal which are capable of impacting tooth survival. Furthermore, the ‘critical’ areas for dentine removal (i.e., the peri‐cervical dentine) have been described but not validated (Clark & Khademi, 2010). A recent study using chairside computer‐aided design and manufacturing (CAD/CAM) technology to measure residual tooth volume failed to demonstrate the inferior survival of structurally compromised teeth that had undergone root canal retreatment (Al‐Nuaimi et al., 2017). However, when the loss of tooth structure was retrospectively analysed within a multifactorial analysis, which also considered endodontic status, periodontal status, as well as several local and general factors, inferior tooth survival was more evident in teeth with greater loss of tooth structure (Al‐Nuaimi et al., 2020).

There is a lack of defined guidance on the most appropriate definitive restoration following the completion of root canal treatment, in particular, which teeth require cuspal coverage (Sequeira‐Byron et al., 2015) and the optimal type of restoration (i.e., full coverage crown or onlay). Furthermore, dilemmas relating to the timing of placement of the definitive restoration (Pratt et al., 2016) are still commonplace. The rapid evolution of new adhesive and ceramic materials (Signore et al., 2009), as well as digital scanning and fabrication technology (Alves de Carvalho et al., 2018) provide clinicians with much greater choice for the restoration of root filled teeth. Digital techniques have become increasingly popular, yet there are few studies to validate these techniques. Dentine bonding has also rapidly developed, but there is a lack of clarity on the optimal materials and techniques for both creating the ideal bonding substrate, as well as the bonding protocol itself. Interestingly, dentine bonding advancements have facilitated a drive to exploit the apparent benefits of these techniques and materials with relevance to emerging concepts such as deep margin elevation, post luting and the adhesive bonding of ceramic restorations. A renewed interest in endocrowns has been driven by the apparent virtues of adhesive bonding which can be achieved with appropriate isolation, restoration and dentine substrate preparation together with well‐executed adhesive cementation.

The rationale for post placement in root filled teeth is still poorly understood and highly subjective. Little consensus on when a post should be used to facilitate core retention exists (Eckerbom & Magnusson, 2001). The innovation of adhesively based post systems (i.e., fibre) has changed the requirements for post dimensions, as well as those for post preparation. These techniques can now be employed within a minimally invasive philosophy and in conjunction with the use of adhesive bonding, permit a greater unity between the endodontic and restorative phases of treatment.

A recent literature review on the restoration of root filled teeth (Bhuva et al., 2021), provides a detailed discussion of the clinical factors influencing the survival of post‐endodontic restorations and teeth following root canal treatment *in vivo*. Therefore, the purpose of this narrative review is to discuss the current status of a number of contemporary concepts and techniques for the restoration of root filled teeth and consider directions for future research.

## SEARCH STRATEGY

For this narrative review, an unrestricted literature search was performed by four evaluators using specified keywords in the PubMed database. Eligibility criteria for included studies required the full text to be available, and to be in the English language, with a publication date up to May 2022. Keywords relating to the restoration of root filled teeth were searched using Medical Subject Heading (MeSH) terms. An additional manual search of references in the included papers was also carried out to identify potentially relevant research. Following the initial screening process, the abstracts of the included papers were read and considered for the suitability, and where relevant, the full text was retrieved.

## STRUCTURAL AND BIOMECHANICAL CHALLENGES OF RESTORING ROOT FILLED TEETH

The structural and biomechanical considerations which affect root filled teeth are complex and diverse and should be considered alongside operative and patient factors (Bhuva et al., 2021; Table 1).

**TABLE 1:** Structural and biomechanical factors affecting root filled teeth.

Structural integrity	Loss of sound tooth volume due to caries, fracture or previous restorative procedures (Ikram et al., 2009)	Access cavity preparation (Reeh, 1989; Pantvisai & Messer, 1995)	Root canal preparation (Hansen & Asmussen, 1993)	Effect of preparation for definitive restoration (Reeh et al., 1989)	
Biomechanical effects	Changes in free water content (Helfer et al., 1972; Sedgley & Messer, 1992)	Collagen alteration (Driscoll et al., 2002; Reddington et al., 2003)	Effect of irrigants and medicaments (Grigoratos et al., 2001; Marending et al., 2007)	Effect of root canal filling materials and techniques (Fuss et al., 2001)	Loss of proprioception (Loewenstein & Rathkamp, 1955; Randow & Glantz, 1986)

These factors, in combination, affect restoration and tooth survival, however, it is not possible to quantify the relative contribution of each variable. That said, it appears that the loss of sound tooth structure is the most critical contributory factor. Evidence also exists for the impact of the ferrule effect (Ferrari et al., 2012), and in combination, the level of root canal treatment difficulty, residual tooth structure, as well as the medical and dental status of the patient (Al‐Nuaimi et al., 2020).

The magnitude of changes to the biomechanical properties of dentine that result from loss of vitality, and the effects of endodontic and restorative procedures, have not been clearly validated. However, a reduction in the free or unbound water content within the porosities found in the dentine matrix and dentinal tubules themselves has been cited as an important reason for the diminished viscoelastic properties of root filled teeth (Kishen & Asundi, 2005; Kishen & Vedantam, 2007; Yan et al., 2017). With the loss of vitality, and a reduction in hydration within the dentine matrix, the size and organization of the collagen fibrils are altered, resulting in loss of plasticity and increased stiffness of the dentine structure (Kishen, 2015). It has been suggested that fully hydrated dentine provides a mechanism to hydraulically dissipate undesirable occlusal and nonocclusal forces away from the root dentine (Pashley, 1990); in the absence of this plasticity, the tooth structure behaves more as a brittle, than tough material (Kishen & Asundi, 2005). These alterations confer increased residual strain and reduced microhardness and resistance to cyclical fatigue, resulting in an increased risk of root fracture (Arola & Reprogel, 2005; Nadeau et al., 2019; Patel et al., 2022).

It is noteworthy to consider the conflicting findings of studies where no changes to the viscoelastic properties of dentine of root filled teeth have been observed (Papa et al., 1994; Sedgley & Messer, 1992). Methodological factors could explain the conflicting results obtained in these laboratory studies. For example, the root filled specimens used in a study by Sedgley and Messer (1992) were stored in saline prior to testing. This prior storage protocol may have permitted rehydration and potential re‐establishment of the viscoelastic properties of the included dentine samples. Similarly, Papa et al. (1994) stored the extracted teeth in aluminium foil until the experimental testing was performed. It has been shown that under normal conditions, 80–85% of dentinal free water loss occurs within 2 h (Jameson et al., 1993). Therefore, the lack of difference observed in the biomechanical performance of root filled teeth in these studies can potentially be explained by the hydration status of the included samples at the time of testing (Patel et al., 2022).

In view of the increased propensity for root filled teeth to undergo microcracks and root fractures, the development of micro‐tissue engineering processes to enhance the biomechanical properties of dentine offers exciting, albeit currently unrealized, potential (Li et al., 2020; Rashidi et al., 2014). Research relating to this area appears to focus on two key areas (Li et al., 2020). First, the induction of additional molecular collagen cross‐links using synthetic and/or natural chemicals could help to overcome some of the undesirable consequences of loss of vitality (Fawzy et al., 2012; Sung et al., 1999). Furthermore, biopolymeric nanofillers can be infiltrated into the dentine matrix to improve its viscoelastic properties (Kishen et al., 2016).

Several laboratory studies have demonstrated improved biomechanical properties of dentine following the incorporation of biopolymeric nanoparticles (Enrich‐Essvein et al., 2021; Li et al., 2020). Proanthocyanidin‐functionalized hydroxyapatite nanoparticles (nHAP_PA) have been used to remineralize and stabilize the collagen matrix of dentine (Enrich‐Essvein et al., 2021). In this study, it was demonstrated that the 1 min application of 15% nHAp_PA increased the flexural strength (MPa) of the included samples. Using nanoindentation, a laboratory study assessed the elastic modulus and hardness of *ex vivo* root dentine samples that had been treated with photodynamically (photodynamic activated) cross‐linked chitosan nanoparticles (CSnps; Li et al., 2020). This process has been reported to produce rapid cross‐linking within the collagen arrangement, initiated by exposure to a photosensitizer with a specific wavelength (Chan et al., 2007; Wollensak & Iomdina, 2009). Essentially, the microtissue engineering of the root canal dentine substrate is the result of CSnps forming a conditioning layer (Kishen et al., 2008, 2016) after which polyanionpolycation ionic complexes are formed (Kishen et al., 2008). The nanoparticles act as hydrophilic space fillers between the collagen fibrils, effectively acting as a plasticizer (Li & Kishen, 2018), thereby improving the flexibility and associated biomechanical characteristics of the treated dentine (Madhavan et al., 2010; Shrestha et al., 2011).

Naturally occurring collagen cross‐linking agents rich in proanthocyanidin have also shown some promise in improving the viscoelastic properties of dentine (Castellan et al., 2011). The results of this study showed that grape and cocoa seed extracts were capable of improving the elastic modulus of dentine by stabilizing collagen matrices through exogenous cross‐linking.

The use of nanopolymeric filler particles to induce collagen cross‐linking and inhibit structural degradation is an interesting area of research that might be applied to root filled teeth to help overcome some of the deleterious effects of loss of unbound water within porosities in the dentine matrix. Further laboratory and clinical research should be carried out to evaluate these exciting concepts.

## STUDY HETEROGENEITY AND CHALLENGES FOR RESEARCH RELATING TO THE RESTORATION OF ROOT FILLED TEETH

The lack of well‐controlled prospective studies relating to the restoration of root filled teeth is primarily due to the unique anatomical, structural and biomechanical considerations for each tooth, as well as the difficulty in both quantifying and standardizing both the assessment methodology and operative protocols with respect to tooth volume loss. In addition to these considerations, the ‘elephant in the room’ appears to be the effect of occlusal factors on the survival of root filled teeth, which is understandably poorly studied, yet may significantly influence the biomechanical performance of root filled teeth. The observations provided by studies on the survival of root filled teeth in relation to tooth location in the arch (Creugers et al., 2005; Fokkinga et al., 2007) and the number of proximal contacts (Aquilino & Caplan, 2002; Caplan et al., 2002; Caplan & Weintraub, 1997), appears to demonstrate a noticeable advantage for nonterminal teeth and those with proximal contacts (Alley et al., 2004; Aquilino & Caplan, 2002). The most striking observation made by Aquilino and Caplan (2002) was that the failure rate for second molars was markedly greater than for any other tooth type, suggesting that the occlusal and nonocclusal forces imparted on these teeth were likely to be a key factor in their inferior survival.

A further indicator of the relevance of occlusal forces is the prevalence of cracked teeth in relation to tooth location reported in the scientific literature, with terminal teeth most frequently affected (Kang et al., 2016; Leong et al., 2020). Furthermore, terminal cracked teeth have been shown to have the poorest prognoses of any, suggesting the same occlusal factors which were responsible for causing the crack initially will also affect the long‐term post‐treatment survival (Kang et al., 2016; Sim et al., 2016; Tan et al., 2006). More than ever, the prevalence of cracked teeth is being acknowledged, and whilst this is at the forefront of endodontic case assessment, there is still a considerable under‐diagnosis of cracked teeth due to a lack of utilization of magnification and coaxial illumination. It is the authors' opinion that the use of magnification with coaxial lighting is incorporated into undergraduate dental training so that from an early stage, students can diagnose, prognosticate, and treat cracked teeth through experiential learning.

A major limitation of many clinical studies relating to the survival of root filled teeth is the relatively short recall period (3–5 years). To compare treatment modalities over this time scale makes it difficult to make meaningful insights into longevity. However, it should also be acknowledged that both prospective and retrospective studies have demonstrated that the majority of restorative, endodontic and/or terminal complications of root filled teeth occur within the first 3 years of initial treatment (Al‐Nuaimi et al., 2020; Salehrabi & Rotstein, 2004). This finding is even more relevant for teeth undergoing root canal retreatment (Kwak et al., 2019). The results of studies with longer recall periods are often adversely impacted by poor recall rates, which may be as low as 28% (Fokkinga et al., 2007), reducing the meaningfulness of the findings.

A further consideration for interpreting clinical data which is based primarily on retrospective analysis is the impact of clinical bias in relation to the choice of definitive restoration (Bhuva et al., 2021). It is more than conceivable that clinicians may elect not to place indirect restorations on teeth with compromised prognoses, whether that be for periodontal, restorative or endodontic considerations. This is one of the most fundamental features of prospective study design, which ensure that selection bias of this type is minimized.

## CURRENT PERSPECTIVES ON RESIDUAL TOOTH STRUCTURE

Evaluating, and more importantly, comparing outcomes of clinical studies relating to root filled teeth is extremely challenging. Several criteria have been used to assess the residual tooth structure; the lack of consistency in the assessment tools across studies makes it very difficult, if not impossible, for systematic reviews or meta‐analyses to combine these data. Furthermore, most of the assessment criteria a qualitative rather than quantitative, and therefore highly prone to bias and subjectivity. The residual tooth structure has been classified qualitatively and quantitively in several ways.

### Ferrule effect

*In vitro* studies relating to the impact of the ferrule effect on the fracture resistance of root filled teeth have shown the improved performance of teeth with adequate supramarginal tooth structure (Ichim et al., 2006; Juloski et al., 2012; Ma et al., 2009; Sorensen & Engelman, 1990). However, clinical outcomes show great variability, although there is still a trend towards improved survival with increased ferrule effect (Cagidiaco et al., 2008; Creugers et al., 2005; Ferrari et al., 2007; Schmitter et al., 2007; Setzer et al., 2011). The outcomes observed in clinical studies are further complicated by the inclusion of post‐retained restorations (Naumann et al., 2018). There are several other factors that need to be considered when evaluating the results of clinical research, which include the definition of the ferrule effect in terms of height (Schmitter et al., 2007) and thickness (Cloet et al., 2017), as well as retrospective study design (Setzer et al., 2011). Due to the difficulties in standardizing prospective research, it is not possible to determine tangible conclusions on the influence of the ferrule effect on the survival of root filled teeth. Meta‐analyses of the available data infer evidence for the benefit of the ferrule effect in premolar teeth, whilst it is not possible to draw such strong determinations for molar teeth (Skupien et al., 2016).

### Number of residual walls

An alternative technique to assess the residual tooth structure which has been used in clinical studies is by evaluation of the residual walls (Cagidiaco et al., 2007; Dammaschke et al., 2013; Mannocci et al., 2002), which may be assessed in terms of the number (Cagidiaco et al., 2008; Ferrari et al., 2012) or percentage (Creugers et al., 2005; Fokkinga et al., 2008; Schmitter et al., 2007). There is significant heterogeneity in what defines a residual wall, and as is the case for the ferrule effect, clinical findings are again affected by confounders. However, despite the limitations of largely retrospective research, the number of residual walls does appear to be an important variable for the survival of root filled teeth. Teeth with no‐ or only one residual wall appear to have reduced survival rates when compared to those with more than one wall (Dammaschke et al., 2013; Ferrari et al., 2012; Nagasiri & Chitmongkolsuk, 2005).

### Residual tooth volume

Although there is currently limited clinical data relating to the impact of residual tooth volume on the survival of root filled teeth, recent studies have utilized digital CAD‐CAM scanning To assess the impact of this variable on the survival of root filled teeth (Al‐Nuaimi et al., 2017, 2020). With this methodology, the issues relating to the variability of interpretation and assessment of the ferrule effect and residual walls are overcome, as all measurements are volumetrically accurate, permitting standardization, not only within the studied sample but also across other studies.

One of the most striking benefits of digital scanning is that residual tooth volume measurements can be made to encompass the ferrule effect/remaining walls in all dimensions and at the same time, the residual coronal dentine. It is the authors' opinion that these nonsubjective, measurable, reproducible and tangible measurements should be utilized further for future prospective clinical outcome studies. Philosophies such as minimally invasive endodontic and restorative techniques could potentially be studied clinically with the use of digital scanning, and furthermore, the potential critical regions for tooth structure removal during endodontic‐restorative procedures were identified.

The existing studies which have used digital scanning, show some correlation between inferior survival and teeth with less than 30% residual tooth volume (Al‐Nuaimi et al., 2017, 2020). The results also provide some interesting insight in respect of more compromised teeth being more susceptible not only to structural but also to endodontic failure (Al‐Nuaimi et al., 2017).

Future research should be directed towards quantitative tooth structure assessment during endodontic‐restorative procedures. As CAD‐CAM scanning evolves, it will hopefully be possible to measure intra‐coronal volumetric tooth structure changes, particularly within the peri‐cervical region of the tooth. It is the authors' opinion that studies assessing the survival of restorations and teeth do not take into consideration of occlusal factors sufficiently within the study design or analysis. Whilst tooth location (Aquilino & Caplan, 2002) and the number of proximal contacts (Aquilino & Caplan, 2002; Caplan et al., 2002; Caplan & Weintraub, 1997) have been studied, the details of occlusion for the included tooth, such as involvement in excursive/protrusive contacts, the presence of working/nonworking side interferences and identification of parafunctional habits could also be included in the preoperative assessment.

## DENTINE SUBSTRATE MODIFICATION AND ADHESIVE BONDING

The fundamental concepts of adhesive bonding include the micromechanical adhesion of composite restorations via etching of enamel, and the creation of an interdiffusional interface of bonding resin and dentine. These protocols have been applied, with no substantial differences, to root filled teeth and those with vital pulps alike. Currently, three‐step dentine bonding agents remain the gold standard in terms of achieving long‐term bonding to dentine (Sauro & Pashley, 2016). Their use is based on the application of a hydrophilic primer to etched dentine, to penetrate the dentinal tubules and the demineralized collagen fibrils, prior to the application of a hydrophobic adhesive based on bisphenol A‐glycidyl methacrylate (Bis‐GMA) and triethylene glycol dimethacrylate (Carvalho et al., 1996). Nevertheless, such bonding strategies remain technique‐sensitive, as clinicians tend to over‐dry the dentine, causing the collapse of collagen fibrils and a consequent lack of resin infiltration within the hybrid layer (Kanca, 1996). The clinical consequences of a lack of infiltration, are marginal discolouration, degradation of the hybrid layer and microleakage, which may then progress into secondary caries (Pashley, 1991; Söderholm, 2007), and potentially cause re‐infection of root filled teeth. Moreover, early degradation of the hybrid layer is even more evident in the now widely used fifth generation of adhesive systems (i.e., self‐priming adhesives), which were developed by combining the primer and adhesive into one solution, to reduce the number of steps necessary to complete the bonding procedure, from three to two (Van Meerbeek et al., 2003).

In the early 2000s, sixth‐generation adhesive systems were developed and identified as ‘self‐etching (SE) primers and adhesives’, Such adhesive systems do not require dentine acid‐etching with phosphoric acid due to the presence of specific functional acidic monomers (i.e., 10‐MDP: 10‐methacryloxydecyl‐dihydrogen‐phosphate) within the formulation of the primer. Such functional monomers can demineralize and prime enamel and dentine substrates simultaneously, which can then be subsequently bonded using separate solvent‐free, relatively hydrophobic adhesives (Pashley et al., 2011; Van Meerbeek et al., 2003).

The latest adhesives are known as ‘all‐in‐one’ and/or ‘universal’ adhesives. These combine etchant, primer and adhesive in a single solution and can be used both with phosphoric acid etching pre‐treatment and as self‐etching adhesives (Pashley et al., 2011; Perdigão et al., 2013). Both self‐etching primers and all‐in‐one adhesives are extensively used in the bonding of composite restorations and cores in root filled teeth, and also in the bonding of fibre and metal posts.

To the best of authors' knowledge, none of the changes to the dentine and enamel substrates that have been associated with loss of vitality or root canal treatment procedures have resulted in a change of bonding strategy. This applies to the loss of free water content (Helfer et al., 1972) and collagen alteration (Driscoll et al., 2002; Reddington et al., 2003) which arguably both contribute to the increased fracture susceptibility of root filled teeth. More recently, several strategies have been suggested to improve bonding to the dentine of root filled teeth, in particular, the use of cross‐linked chitosan nanoparticles was found to reduce the degradation of dentinal collagen and improve the stability of the adhesive interface (Xiong et al., 2020).

Several promising strategies have been advocated to reduce the stress concentration at the bonding interface that usually occurs during the light‐curing of resin composites, all of which have the potential to increase the longevity of both direct and indirect composite restorations. Unfortunately, to the best of authors' knowledge, none of these strategies have gone past initial clinical trials (Nikolaenko et al., 2004).

The use of ‘stress‐absorption’ resin flowable liners or glass‐ionomer cements employed as the base and/or dentine‐substitute materials (Irie et al., 2004) may attenuate the polymerization stresses generated at the dentine‐bond interface, reducing the risk of gap formation, microleakage and secondary caries (Sampaio et al., 2011; Sauro et al., 2018). This is of particular importance when nonaxial stresses are generated during parafunction (e.g., bruxism), and which in turn, may significantly affect the integrity of the bonding interface (Khvostenko et al., 2015; Toledano et al., 2015).

The use of air‐abrasion systems in combination with aluminium oxide or bioactive glasses to prime dentine following endodontic treatment, and prior to restorative procedures, will help to remove root filling material/sealer residues and provide smoother dentine walls with rounded internal line angles; this may reduce the stress concentration along the bonding interface due to a reduced C‐factor (Banerjee, 2013; Spagnuolo et al., 2021) and also reduce crack propagation and the probability of fatigue failure (Ayad et al., 2011). Furthermore, when performing dentine air‐abrasion using bioactive glass, a ‘bio‐reactive’ smear layer is produced on the dentine surface, which is then incorporated within the bonding interface created using resin‐modified glass ionomer cements or SE adhesives. Such bioactivity is due to the hydrated silica Si(OH)_4_ produced by bioglasses when in contact with water or saliva, and may stop the degradation processes at the bonding interface (Sauro, Watson, Thompson, & Banerjee, 2012b; Sauro, Watson, Thompson, Toledano, et al., 2012a). The bio‐reactive layer condensates within the demineralized dentine collagen (Pashley, 1991) fossilizing the dentine proteases (e.g., metalloproteinases (MMPs)) and serving as a template for the precipitation of Ca^2+^ and PO_4_^3−^ which may remineralize and protect the hybrid layer. Moreover, the alkaline pH generated by bioactive glasses, may also have antibacterial properties and reduce the risk of secondary caries (Bauer et al., 2019).

Other methods advocated to preserve the durability of dentine‐bonded interfaces include the pre‐treatment (1 min) of acid‐etched dentine using chlorhexidine (2% CHX) before bonding. Indeed, *in vitro* (Yiu et al., 2012) and *in vivo* (Carrilho et al., 2007) studies have demonstrated that CHX may inhibit the action of several types of MMPs, as well as dentinal cysteine cathepsins, which cause degradation of the hybrid layer (Scaffa et al., 2012).

Quaternary ammonium compounds (QACs) can be employed to reduce the enzyme‐mediated collagen degradation within the hybrid layer. These are molecules with lower molecular weight than CHX, which may easily infiltrate the demineralized dentine, leading to a more reliable anti‐MMP effect within the hybrid layer (Pupo et al., 2014). Benzalkonium chloride is a QAC with potent antibacterial properties that have been advocated as a potential anti‐proteolytic agent to be used before bonding procedures (Sabatini & Patel, 2013). However, CHX was found to be more efficient in inhibiting MMPs and cathepsin‐K than 2% benzalkonium chloride (Imazato et al., 2006). Moreover, light‐curable QAMs such as methacryloyloxydodecylpyridinium bromide have also shown encouraging results in reducing the proteolytic degradation of dentine‐bonded interfaces, as well as, inhibiting bacterial growth and reducing the risk of secondary caries (Sauro, Watson, Thompson, & Banerjee, 2012b). A further useful approach to reducing the proteolytic action of MMP‐2 and MMP‐9 is based on the use of materials able to release fluoride (F−) ions (Feuerstein et al., 2007).

Whilst the above considerations for adhesive bonding may not have been shown to influence clinical outcomes, it is important to appreciate that optimal bonding may be more relevant to modern treatment concepts such as minimal preparation onlay restorations, adhesive post cementation and deep margin elevation.

## COMPOSITE RESIN MATERIALS

Composite resin is frequently used as both a definitive restorative and core material following root canal treatment. The development of self‐adhesive resins and bulk‐fill materials, for placement within the pulp chamber, has provided greater applicability and reduced technique sensitivity (Hayashi et al., 2019).

There are a number of challenges to placing composite resin materials for core, or definitive restoration, specific to root filled teeth. Importance of removing remnants of root filling materials and sealer residues (Mannocci et al., 2008) is paramount. The use of fine ultrasonic tips with copious water spray may be of great benefit for this purpose. Angled, endodontic microsurgical tips may be of particular use in minimally invasive access cavities in molar teeth, as they can be used to clean the undercuts within the pulp chamber (Chan et al., 2022).

**FIGURE 1:**
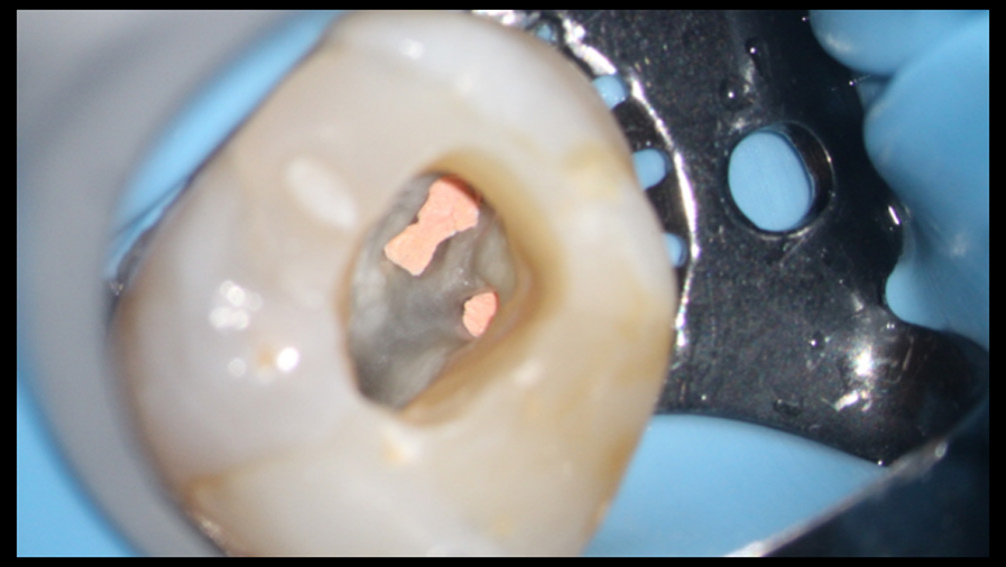
Pulp chamber preparation following completion of the endodontic treatment and prior to placement of composite resin core. The dentine surface has been cleaned with ultrasonics, after which alcohol has been used to sequester residual eugenol from the pulp chamber. Air‐abrasion in combination with aluminium oxide or bioactive glasses may also be used.

A further consideration is the use of eugenol‐based root canal sealers and/or temporary materials. This phenolic compound has been reported to have a detrimental effect on the adhesion of resin materials to dentine (Menezes et al., 2008; Schwartz et al., 1998). Resin composites polymerize by the addition of free radicals. However, this process may be inhibited by eugenol (2‐methoxy‐4‐allyphenol) and is capable of penetrating into root canal dentine (Kielbassa et al., 1997). In addition to the use of ultrasonics, and as discussed earlier, air‐abrasion may be a useful adjunct to improve the dentine substrate prior to bonding. Furthermore, if eugenol‐based root canal sealer and/or temporary materials have been used, polymerization inhibition of any composite resin materials may be reduced by rinsing the dentine with isopropyl alcohol to sequester any accessible free eugenol (Tian et al., 2021; Figure 1).

**FIGURE 2:**
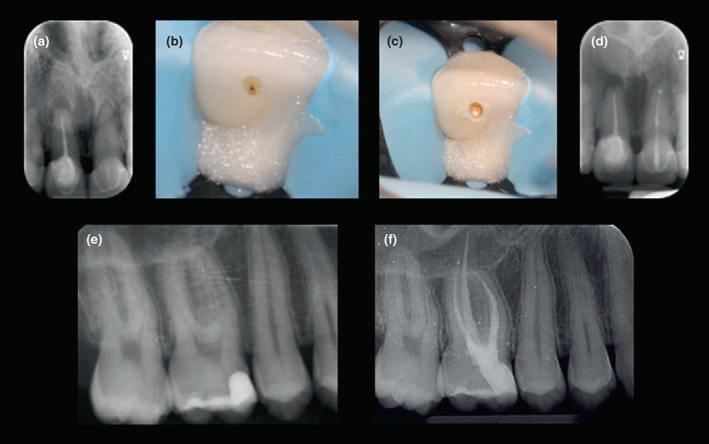
The placement of core materials in minimally invasive access cavities presents a challenge (a–d) preoperative periapical radiograph, intra‐operative images and post‐treatment radiograph of minimally invasive root canal treatment of 21. Predictable light transmission for use of conventional composite resin was not possible during core placement so a dual‐cure material was used in this case (e, f) ‘restoratively‐driven’ access during root canal treatment of 16. The diligent use of magnification, an elongated delivery tube to place the composite resin (e.g., Accudose; Centrix) permitted placement of the restoration without void formation.

The placement of composite resin materials within the root canal space and the pulp chamber is complicated by the limited access for both restoration placement and light transmission. This may lead to void formation and/or incomplete polymerization of the composite resin materials. Employing a minimally invasive endodontic philosophy will make composite core placement even more challenging (Figure 2). This has been highlighted in studies demonstrating that the percentage of voids observed within the composite restoration is greatest in teeth with minimal access cavities (Pereira et al., 2021; Silva et al., 2020). In these studies, it was also demonstrated that the use of bulk‐fill, rather than conventional composites, led to less void formation. The development of bulk‐fill materials has been rapid, with the manufacturers of these materials reporting increment depths of up to 10 mm, thereby, facilitating much shorter procedural times.

To permit larger increments of material to be placed, the manufacturers of bulk‐fill materials have used several strategies to increase the depth of cure which include reducing filler content (Ilie et al., 2013) and increasing particle size (Ilie et al., 2013) and the addition of photoinitiators. Furthermore, the shrinkage of these materials has been reduced by incorporating shrinkage stress modulators into the compositions (Isufi et al., 2016). For example, SDR (Dentsply Sirona) utilizes a modulator that interacts with the camphorquinone photoinitiator during polymerization to reduce the speed of elasticity modulus development. Although the depth of cure of bulk‐fill materials is good, it should also be considered that curing light intensity will diminish with distance. As the base of an endodontic cavity may be several millimetres from the light source, it may be prudent to consider smaller initial increments to offset the increased curing distance (Prati et al., 1999; Rueggeberg et al., 1993). As a result, with the exception of dual‐cure compositions, bulk‐fill composite materials should not ideally be used in increments exceeding 4–5 mm.

A problem for all composite resin materials at the microscopic level is gap formation at the bonding interface (Benetti et al., 2015). Gaps can occur for several reasons, such as insufficient adhesion at the tooth‐restoration interface due to polymerization shrinkage, adhesive resin degradation as a result of insufficient light‐curing, fatigue caused by ageing, differences in the thermal expansion coefficient of the tooth substrate and composite resin, or insufficient material placement (Moszner et al., 2008).

**FIGURE 3:**
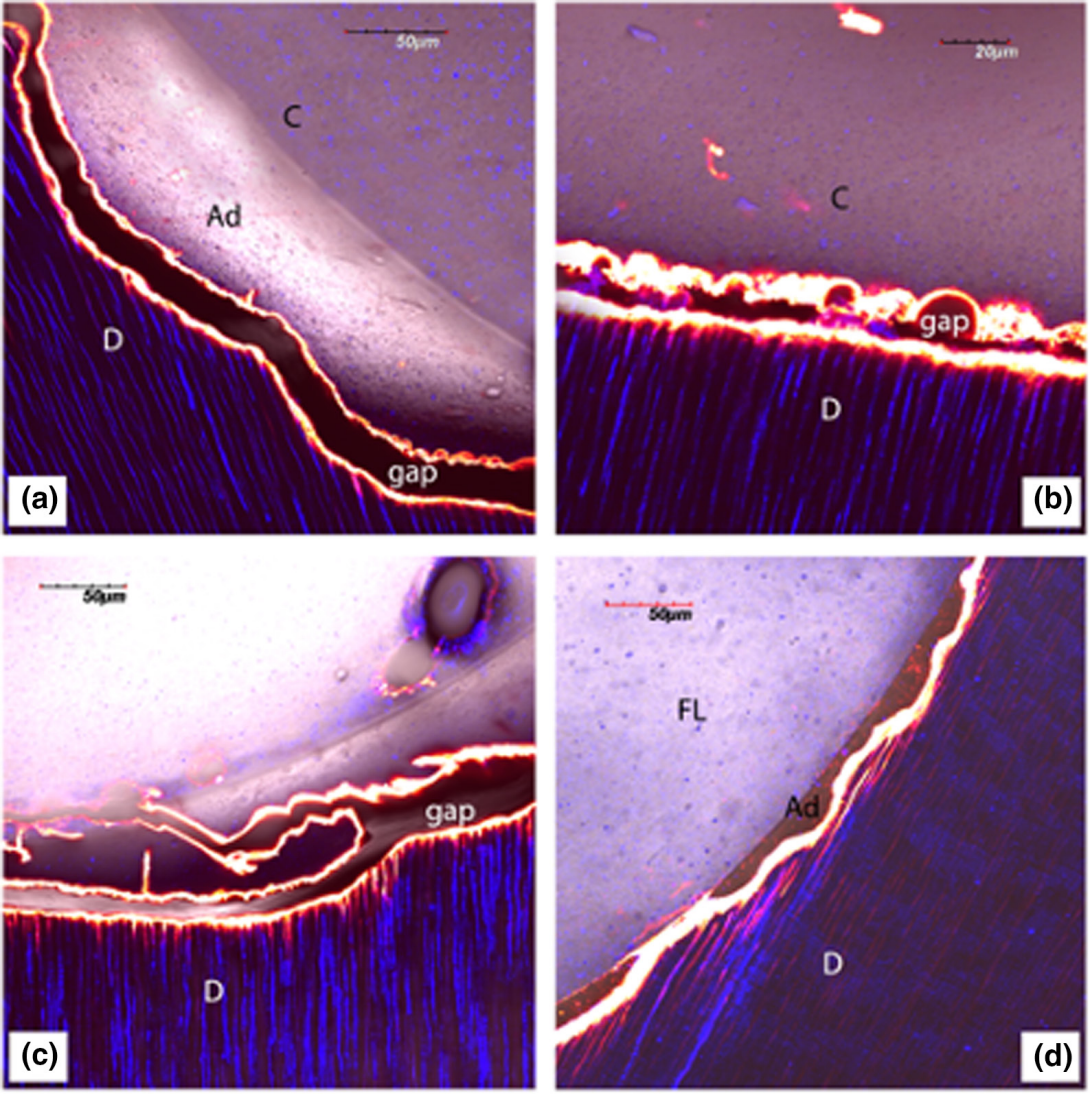
Dye‐assisted confocal microscopy of resin‐dentine interfaces created using different bonding and restorative procedures performed in class 1 cavities subsequent root canal treatment *in vitro*. (a) Single‐projection images of the resin‐dentine interface created by the application of a bulk‐fill composite (Filtek One Bulk‐Fill; 3M ESPE AG) following the use of a universal adhesive (Scotchbond Universal; 3M ESPE AG) in self‐etching (SE) mode. It is possible to see a clear gap between the dentine (d) and the adhesive/composite (Ad/C) most likely due to polymerization shrinkage, which has caused the debonding in adhesive mode (b). (c) Further images of resin‐dentine interface, following conventional composite (Filtek Supreme XTE; 3M ESPE AG) placement with the use of a universal adhesive in SE mode. Once, again it is possible to see a clear gap between the dentine (d) and the adhesive/composite (Ad/C) due to polymerization shrinkage. (d) Single‐projection images of resin‐dentine interface created by the application of a flowable ‘bioactive’ restorative composite (ACTIVA Restorative; Pulpdent) following the use of a universal adhesive in SE mode. In this case, gap formation between the dentine (d) and the adhesive/composite (Ad/C) is much less evident. This may be due to the mechanical and compositional characteristics of the material (i.e., resin‐modified glass ionomer cement containing modified calcium phosphates), which are proposed to create less stress on the bonding interface, particularly when left undisturbed for a couple of minutes prior to light‐curing.

Hayashi et al. (2019) demonstrated that light‐cured bulk‐fill resin composites had varying degrees of gap development and shrinkage within a 4‐mm deep cavity (Figure 3). Furthermore, it has been reported that high‐viscosity bulk‐fill composites are associated with greater gap formation volumes than low‐viscosity bulk‐fill materials (Oglakci et al., 2019). The authors also demonstrated that using an resin‐modified glass‐ionomer cement (RMGIC) liner reduced gap formation volume significantly in high‐viscosity bulk‐fill composites. Although manufacturers claim that bulk‐fill composites have less polymerization shrinkage than traditional composites, there is insufficient literature on the influence of intermediate lining materials (Alomari et al., 2001).

**FIGURE 4:**
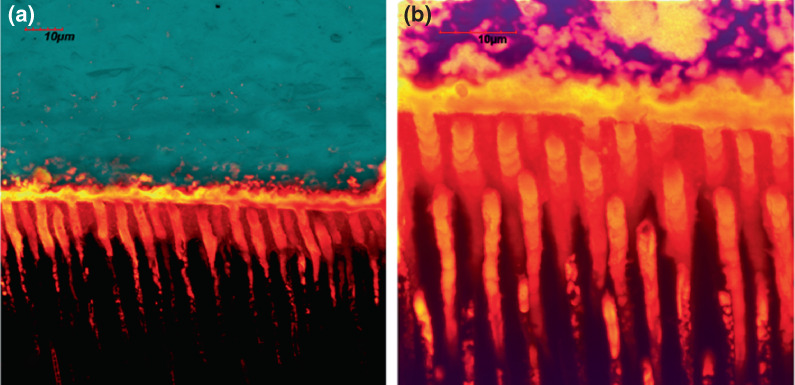
Dye‐assisted confocal microscopy of resin‐dentine interfaces created using an experimental self‐etching adhesive applied on dentine previously air‐abraded with bioactive zinc‐doped bioglass powder after 3 months of storage in artificial saliva. (a): Single‐projection images of resin‐dentine interface that was immersed in 0.5 wt% calcium‐chelating dye solution 26 (Xylenol Orange; Sigma–Aldrich) after maintaining the specimens for 3 months in artificial saliva (AS). It is possible to observe, especially at higher magnification (b), the presence of a clear calcium‐based mineral deposition within the resin‐dentine interface and inside the dentine tubules induced by the bioactive glass.

The use of flowable composites or RMGIC as liners or dentine substitution materials, has been reported to provide a ‘stress‐absorption’ effect at the bonding interface (Irie et al., 2002, 2004) and to decrease gap formation, microleakage and deterioration over time (Kakaboura et al., 2007; Sampaio et al., 2011; Figures 3 and 4). Moreover, it has been recently reported that the use of modern ion‐releasing materials such as conventional RMGIC or RMGIC‐based composite (ACTIVA restorative; Pulpdent) used as dentine replacement materials may preserve the *in vitro* bonding performance of modern universal adhesives bonded to dentine (Sauro et al., 2019; Slimani et al., 2021). However, further studies are required to validate their use.

Practical considerations for the use of bulk‐fill materials include the placement and pooling of the adhesive bonding resin. Fine microbrushes (Microbrush X; Young Innovations Europe GmbH) are useful for this purpose, as are paper points, which may also be used to absorb surplus bonding agents. The length of the delivery tip of proprietary composite compules may not be of adequate length to reach the base of the access cavity, increasing the risk of void formation. This may be overcome using elongated needle delivery tubes (e.g., Accudose; Centrix); these can either be filled with the desired material or placed over the proprietary compule to provide deeper access.

Despite concerns that relate to composite bonding in general, bulk‐fill composite materials should still be considered a good choice for post‐endodontic core placement, due to their favourable properties and handling.

## DEEP MARGIN ELEVATION

The occurrence of subgingival proximal margins due to caries or previous restorative procedures is a common clinical challenge encountered during endodontic‐restorative procedures. The ‘threshold’ for deeming a tooth unrestorable varies significantly amongst clinicians. It is critical that the restorability of a tooth is established prior to endodontic treatment to avoid difficulties later in the treatment process. Embedded in convention, there has often been a dissociation between the endodontic and restorative phases of treatment, leading to poor treatment planning decisions, poor asepsis during root canal treatment and suboptimal restoration placement. Moreover, teeth that have undergone root canal treatment may later be deemed unrestorable. However, it is promising to see emerging trends, not only in clinicians increasingly taking holistic ‘ownership’ of both the endodontic and restorative aspects of treatment but also in the level of difficulty of cases being undertaken. It is the authors' opinion that restorative treatment should be an integral part of the skillset of clinicians undertaking endodontic treatment.

Historically, the management of subgingival restorative margins has involved crown lengthening procedures which comprise surgical osseous and soft tissue repositioning to establish supragingival margins, and to permit definitive restoration without impinging on the biological width. However, these procedures require an additional surgical procedure, time and cost to the patient, and will reduce the bone support of both the treated and neighbouring teeth. Furthermore, the procedure can often be complicated in the interproximal region by a lack of adequate space between the adjacent teeth.

An alternative approach to managing the restoration of deep interproximal margins is the deep margin elevation (DME) or cervical margin relocation concept which was first described by Dietschi and Spreafico (1998). This involves the relocation, or elevation, of the subgingival margin to a more coronal position using direct composite resin. The fundamentals of this procedure rely on optimal rubber dam isolation, the use of appropriate and innovative matrix systems/techniques and an optimal bonding strategy (Magne, 2021). One of the main concerns with bonding below the cementoenamel junction is that the marginal seal will be entire with dentine and/or cementum. Achieving these objectives with optimal isolation is a technique‐sensitive challenge (Van Meerbeek et al., 2005). It is therefore fundamental to understand the importance of developing the necessary skills in isolation, matrix placement and bonding protocols to optimize outcomes, moreover, when these objectives cannot be fulfilled, surgical crown lengthening may be a valid alternative.

Clinical data relating to the performance of teeth restored with DME techniques are lacking, with a systematic review on the subject highlighting that existing research is almost entirely limited to *in vitro* studies and clinical case reports (Juloski et al., 2018). There are limited clinical studies on the long‐term outcomes of DME, and the results should be interpreted with caution due to the lack of data specific to root filled teeth. A study by Bresser et al. (2019) followed 197 indirect restorations (including 45 endodontically treated teeth) with DME up to 12 years, with a mean follow‐up of 57.7 months. The cumulative 12‐year survival rate was determined to be 95.9%, with the majority of failures due to recurrent caries. However, a significantly higher incidence of tooth and restoration fractures occurred in root filled teeth when compared with those with vital pulps. Further case series have demonstrated excellent survival rates between 5 and 21 years (Dietschi & Spreafico, 2019; Ghezzi et al., 2019), however, the included numbers are small, and therefore, should be interpreted with caution.

A concern regarding the DME technique is the potential violation of the biological width (Broadbent et al., 2006) and the associated risk of periodontal inflammation and attachment loss (Kamin, 1989). Results in relation to the impact of DME on periodontal health demonstrate conflicting results (Ferrari et al., 2018; Sarfati & Tirlet, 2018). Ferrari et al. (2018) assessed the health of the periodontal tissues of 35 posterior teeth restored with either the DME technique or shoulder preparation after 12 months. They found a significantly higher incidence of bleeding on probing in the DME group, and this was most prevalent in teeth where the distance between the cavity margin and the crestal bone was 2 mm or less. However, other authors have reported favourable periodontal responses to DME (Ghezzi et al., 2019; Sarfati & Tirlet, 2018). A further study demonstrated no differences in clinical or histological findings of the periodontal tissues adjacent to the DME when compared with untreated controls (Bertoldi et al., 2020). There is also differing opinion on the acceptable depth of the DME, and specifically, the proximity of the restoration margin to the connective tissue and the crestal bone (Castelo‐Baz et al., 2021; Ghezzi et al., 2019; Sarfati & Tirlet, 2018). Proponents of DME have suggested that the technique may be utilized at any depth in relation to the crestal bone, as long as optimal rubber dam isolation can be achieved (Ghezzi et al., 2019), and that a shorter long junctional epithelial attachment can be maintained without inducing periodontal attachment loss. Intuitively, in cases with deep proximal caries, attachment loss to the base of the carious lesion has already occurred, and where the alternative of surgical crown lengthening will lead to further attachment loss, it appears sensible that this may be a valid technique. However, it is necessary for prospective clinical research to validate the long‐term stability of DME in root filled teeth.

Techniques such as DME should be considered necessary skills for those carrying out endodontic‐restorative treatment. The ability to isolate and restore deep margins will permit the retention of many previously condemned teeth. By restoring these areas prior to performing the endodontic treatment, several objectives are achieved; these include restorability being established, attainment of optimal isolation during the endodontic treatment and ease of preparation of the definitive restoration. Importantly, the use of DME will also facilitate both the preparation and adhesive luting of indirect restorations with subgingival proximal margins, which otherwise, would need to be restored with conventionally cemented indirect restorations, which in turn require greater tooth reduction (Juloski et al., 2018).

## ALL CERAMIC CROWNS AND ONLAYS

The evolution of all ceramic materials for the provision of indirect restorations has led to their routine use for both root filled teeth, and those with vital pulps. These materials provide huge aesthetic advantages but with little compromise to restoration strength and longevity. Numerous materials have been developed to produce all ceramic restorations; these include conventional or traditional feldspathic porcelain, aluminous porcelain, glass infiltrated alumina, zirconia, glass ceramic, reinforced glass ceramic (leucite and lithium‐disilicate) and densely sintered alumina. Unfortunately, there is a sparsity of data, with little available research assessing the performance of all ceramic restorations specifically on root filled teeth (Dioguardi et al., 2021); this should be considered highly relevant to the interpretation of the results of the available studies (Morimoto et al., 2016).

**FIGURE 5:**
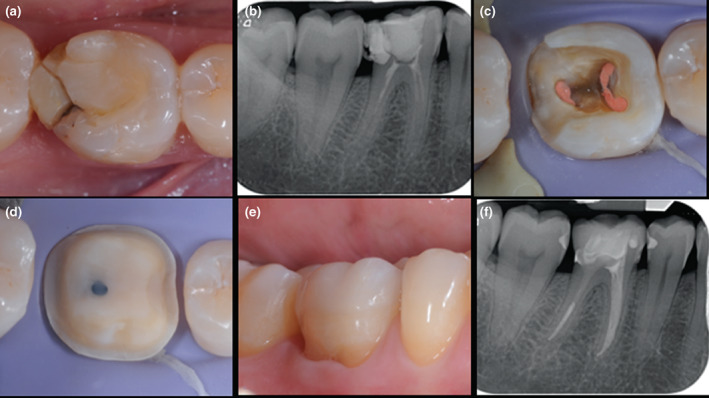
Lithium disilicate ceramic onlay (IPS e.max, Ivoclar Vivadent) placement following root canal retreatment of 36 (a, b) preoperative occlusal view and long‐cone periapical radiograph (c) cleaned and prepared dentine surface prior to deep margin elevation and core placement (d) completed core and onlay preparation (e, f) post‐treatment occlusal view and postoperative periapical radiograph.

**FIGURE 6:**
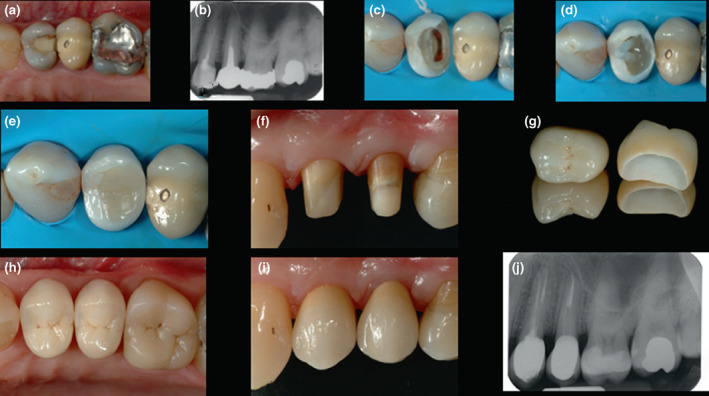
Zirconia crown (Lava; 3M ESPE AG) replacement 25 and crown placement 24 following root canal retreatment (a, b) preoperative occlusal view and long‐cone periapical radiograph (c) completed root canal retreatment and dentine surface preparation (d, e) pre‐endodontic build up, fibre post and core placement (f) completed full‐coverage crown preparations (g) crowns prior to cementation (h, i) finished restorations with postoperative occlusal and buccal views (j) follow‐up periapical radiograph at 10 years.

A systematic review evaluating the survival and complication rates of various all ceramic and metal ceramic crown restorations found that they showed comparable survival rates at 5 years (Sailer et al., 2015). A total of 9434 all ceramic and 4663 metal ceramic crowns were included, however, the number of root filled teeth and those with vital pulps was not specified. It was found that for posterior teeth, densely sintered alumina (Procera; Nobel Biocare, Zürich‐Flughafen, Switzerland) and reinforced glass ceramic crowns (IPS Empress, IPS. e.max; Ivoclar Vivadent, Schaan, Liechenstein; Figure 5) performed similarly to metal ceramic crowns. Glass ceramic (DICOR; Dentsply Sirona) and In Ceram (Vita Zahnfabrik) crowns had lower survival rates when placed on premolar and molar teeth. Posterior all ceramic crowns had more failures than anterior all ceramic crowns. The most common modes of failure for all ceramic crowns collectively were ceramic chipping, framework fractures and loss of vitality (biological failure). The cumulative 5‐year survival rates were 95.7%, 96.6%, 94.6% and 96% for metal ceramic, leucite or lithium disilicate reinforced glass ceramics, glass infiltrated alumina and densely sintered/alumina crowns, respectively. The authors concluded that leucite, lithium disilicate reinforced glass ceramic or alumina‐based oxide all ceramic crowns could be recommended as an alternative to gold‐based metal ceramic crowns for both anterior and posterior teeth. Feldspathic and silica‐based ceramics were associated with higher failure rates when used for the restoration of posterior teeth. Furthermore, layered zirconia‐based crowns were considered inferior due to loss of retention and fracture of the ceramic veneering. However, in other studies zirconia crowns were shown to have equivocal veneering fractures to other restorations and remain a popular choice with clinicians (Laumbacher et al., 2021; Figure 6).

A prospective study assessed the longer‐term survival and complication rates for lithium disilicate e.max crowns (Teichmann et al., 2017). The authors assessed the 10‐year outcomes for 106 all ceramic crowns and observed relatively low survival and chipping‐free rates of 86.1% and 83.4%, respectively. In this study, there were fairly even proportions of restorations that were adhesively and conventionally luted; a rubber dam was used for cementation where possible. It was observed that the 5‐year chipping rate was relatively high and it may be that the cementation process is relevant to the biomechanical performance of these restorations.

A key attribute of all ceramic restorations is that they can be adhesively bonded to dentine, although this requires appropriate substrate preparation (D'Arcangelo et al., 2014), and disciplined bonding protocol (Santos Jr et al., 2009) and good moisture control with a rubber dam. Adhesive cementation permits minimal preparation techniques to be employed, such that there is less reliance on creating resistance and retention form within the residual tooth tissue. This facilitates both the preservation of residual tooth structure and the restoration of compromised teeth that may not be possible with conventional preparation techniques and materials. Furthermore, using an adhesively bonded technique there is less requirement for intra‐coronal core retention, and as such, the use of posts. It is for these reasons, that indirect onlay restorations have increased in popularity in recent times, in line with minimum intervention endodontic‐restorative philosophies. However, as discussed earlier in this review, it is imperative to acknowledge the differences in bonding for root filled teeth when compared with those with vital pulps, due to the ultrastructural differences in the dentine substrate and free water content (Abo‐Hamar et al., 2005; Öztürk et al., 2013; Rosa et al., 2015), as well as the effects of the endodontic procedures themselves (Abad‐Coronel et al., 2019). The impact of these considerations has not been determined in clinical studies.

There is a considerable lack of data relating to the performance of all ceramic onlays, rather than full coverage crowns, on root filled teeth. However, Ferrari et al. (2019) carried out a randomized clinical trial, evaluating the survival of lithium‐disilicate onlays on root filled premolar and molar teeth with a 3 year follow‐up period. The parameters of the onlay preparations were standardized by the authors with occlusal reduction limited to 1.0–1.5 mm. Restoration and tooth survival rates of 93.3% for premolars, and up to 100% for molars with 50% or more coronal residual tooth structure (after preparation) were demonstrated. There were no significant differences observed in relation to tooth type (premolars or molars) or fibre post placement, although the failure risk was slightly higher for premolars. Despite the prospective and randomized study design, the limitations of the relatively short observation period and the exclusion of patients with heavy occlusal contacts and/or evidence of parafunctional habits should be taken into consideration.

An observational study carried out by a single operator in private practice assessed the long‐term survival of 2392 pressed acid‐etched e.max lithium disilicate glass ceramic complete and partial coverage restorations in posterior teeth (Malament et al., 2021). Of these restorations, 1782 were full‐coverage crowns, whilst 610 were onlay designs. All restorations were etched with hydrofluoric acid, and then silanated, at the time of cementation. The estimated cumulative survival at 16.9 years for all restorations was 96.49%, with no significant differences observed between the full coverage crowns and onlays. Unfortunately, the authors do not provide any information on the numbers of root filled or those with vital pulps included in the study. However, the number of restorations included, as well as the relatively long follow‐up is useful for evaluating patient‐centered outcomes. Interestingly, there was no difference in survival when the restorations had a thickness of less than 1 mm. Other studies have suggested that lithium disilicate ceramic onlays may have adequate fracture resistance in thicknesses of 0.5–1.0 mm (Guess et al., 2013). This is an important observation, which requires further investigation to ascertain whether more conservative preparations may be considered to maximize the preservation of residual tooth structure. Such minimal preparations would also facilitate the possibility of greater enamel bonding, particularly when used in conjunction with a minimally invasive access cavity preparation concept. It may be argued that for such restorations, the additive value of improved bonding to the residual tooth structure achieved by retaining occlusal enamel, may in itself, be justification for a minimally invasive endodontic‐restorative philosophy.

## CAD‐CAM RESTORATIONS

CAD‐CAM cuspal coverage restorations have significant potential to expedite the restoration of root filled teeth through same‐visit chairside restoration, however to date, evidence is lacking as to whether CAD‐CAM restoration *per se* improves treatment outcomes (Carvalho et al., 2018). As is the case for all indirect restorations, the available data does not specifically detail the performance of CAD‐CAM restorations on root filled teeth. Thus, there is a need for prospective well‐designed clinical trials to answer key questions about the relative outcomes and optimal protocol for the CAD‐CAM restoration of root filled teeth. A systematic review followed 2916 single‐unit CAD‐CAM indirect restorations for a mean period of 7.0 years (Alves de Carvalho et al., 2018). Of the included restorations, 1826 were either onlays or inlays, with an estimated 5‐year survival rate of 90.9%.

**FIGURE 7:**
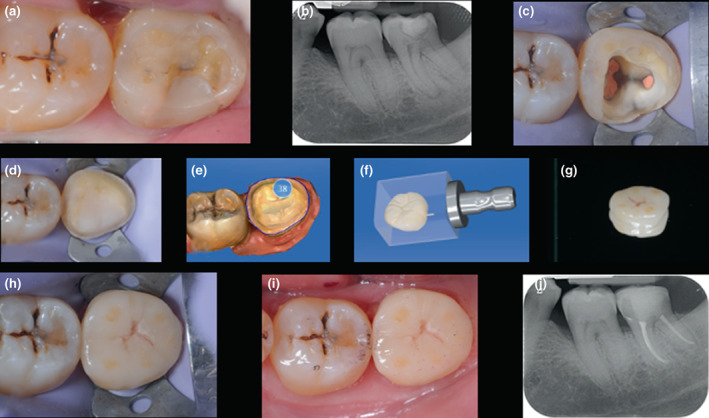
Lithium disilicate onlay (IPS e.max CAD) placement following root canal treatment of symptomatic 38 (a, b) preoperative occlusal view and long‐cone periapical radiograph (c) completed root canal treatment and dentine surface preparation (d) completed core and onlay preparation (e–g) the onlay is fabricated using computer‐assisted design and manufacturing (CAD‐CAM) (h, i) finished restoration with postoperative occlusal view and follow‐up periapical radiograph (j).

There are three key outstanding questions regarding the adoption of CAD‐CAM technology. First, adopting a CAD‐CAM‐based workflow can lead to bias amongst clinicians towards prescribing indirect rather than direct restorations, given the relatively high financial outlay involved in purchasing the scanning and/or milling hardware. This influence on decision‐making may not always lead to superior outcomes for root filled teeth. Mannocci et al. (2002) demonstrated that post‐endodontic fibre post‐retained composite restorations were as successful at 3 year in class II premolar cavities, as those restored with full coverage crowns. However, in many cases, an indirect restoration with cuspal coverage provides better outcomes (Pratt et al., 2016). The second question is regarding timing, as the key advantage to being gained from CAD‐CAM is that restorations can be completed expediently after endodontic treatment, perhaps even at the same visit as the root canal treatment (Figure 7). Whilst evidence is lacking on single‐visit endodontic‐restorative treatment there is retrospective data showing that timely indirect restoration provides better outcomes; Pratt et al. (2016) reported that teeth that were restored with crowns more than 4 months after root canal treatment were almost 3 times more likely to get extracted compared when compared with teeth that received crowns within 4 months of root canal treatment. The third key question is regarding the material choice for CAD‐CAM restorations, namely ceramic versus hybrid composite/ceramic. Hybrid composite is becoming increasingly popular due to its optimal machining properties, however, prospective data on CAD‐CAM hybrid ceramic restorations for root filled teeth are lacking. The primary mode of failure of CAD/CAM restorations is fracture; reasons for fracture of ceramic restorations may include low flexural strength of the material, subsurface flaws of CAD‐CAM ceramics produced during machining, insufficient polishing of the occlusal surfaces after adjustment and parafunctional habits, with equivalent rates being shown for hybrid ceramics in early data (Lu et al., 2018). However, a key issue with CAD‐CAM hybrid ceramics is their failure in full crown scenarios, due to the excessive hoop stresses that occur, and therefore, many manufacturers limit their indications for partial coverage restorations and contra‐indicate their use for full coverage crowns (Bomfim et al., 2020).

## INDIRECT COMPOSITE RESIN RESTORATIONS

Resin composite materials for indirect restoration consist of a polymeric matrix reinforced by fillers which may be inorganic (ceramic, glass or glass ceramic), organic or composite (Ferracane, 2011). Unfortunately, the original resin blocks suffered from increased resin wear, loss of surface polish and colour instability (Douglas, 2000). In recent times, the new formulations of so‐called ‘resin‐matrix ceramics’ for CAD‐CAM combine the advantageous properties of ceramics, such as colour stability and durability, with those of composite resin, such as low abrasiveness and improved flexural properties. These materials have been sub‐classified as polymer‐infiltrated ceramic networks (e.g., VITA Enamic; VITA Zahnfabrik) or resin‐based composites (e.g., Cerasmart; GC Corporation; Spitznagel et al., 2018). Despite the evolution of these materials, concerns remain as to their long‐term wear and fracture resistance, as well as marginal discoloration (Albelasy et al., 2020; Tekçe et al., 2016). In particular, there is concern regarding their strength in areas of high functional and nonfunctional stresses (Morimoto et al., 2016). However, significant advantages of these resin materials are the ease of fabrication and the ability to service the restorations intra‐orally.

The long‐term survival of indirect composite restorations has not been evaluated; a recent systematic review has highlighted the need for longer‐term prospective research (Fathy et al., 2022). The success and survival of 103 CAD‐CAM adhesively bonded polymer‐infiltrated ceramic network posterior onlays and inlays were prospectively followed for 3 years (Spitznagel et al., 2018). The authors reported a survival rate of 95.6% and a success rate of 82.4% for onlay restorations at the end of the follow‐up period. The main complications observed were deterioration in marginal adaptation and colour, together with increased surface roughness; the majority of the teeth in this study were those with vital pulps. The medium‐term survival of indirect composite resin onlays placed on root filled teeth has been retrospectively assessed by Chrepa et al. (2014). A total of 189 restorations (31 premolars and 159 molars) were evaluated over a median follow‐up time of 37 months. Restoration survival was reported to be 96.8% at the end of the follow‐up period. The findings of this study appeared to show a lower complication rate in respect of marginal adaptation and discolouration. Overall, these studies demonstrate excellent medium‐term outcomes, which are equivocal, but the time‐dependent trends of marginal breakdown are a concern for long‐term restoration stability and as such, further evaluation is required.

## POSTS

Root canal posts primarily provide retention for the coronal restoration of substantially compromised root filled teeth. Even with the improvement of adhesive luting techniques, the contribution to the stability of the root by adhesively placed root canal posts remains questionable and is predominantly considered for the restoration of weakened traumatized maxillary anterior teeth with thin dentinal walls (Krastl et al., 2021; Ree & Schwartz, 2017). The amount of residual coronal tooth structure and the tooth type are key factors in determining the need for a post. Post placement is recommended for root filled teeth with no remaining coronal walls (Naumann et al., 2018) and those with one remaining wall (Ferrari et al., 2022). Posterior teeth with adequate depth and shape within the pulp chamber for core retention can be reliably restored without posts (; Ferrari et al., 2019). Biomechanical considerations lead to the assumption that posts are more frequently needed for maxillary anterior teeth due to the higher risk of mechanical failure in this region (Schmitter et al., 2011; Torbjörner & Fransson, 2004). However, a recent meta‐analysis revealed similar failure rates with short‐ to medium‐term follow‐up of post‐ and core restorations in anterior and posterior teeth (Garcia et al., 2019).

**FIGURE 8:**
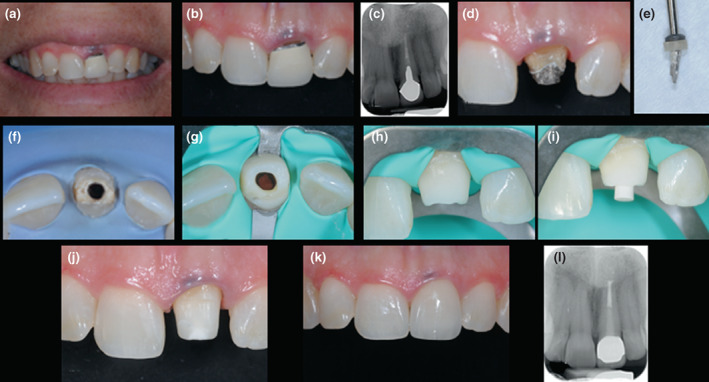
Zirconia crown (Lava; 3M ESPE AG) replacement following root canal retreatment 21 (a–c) preoperative buccal views and long‐cone periapical radiograph (d–f) removal of cast post and root canal retreatment (h–j) internal bleaching followed by fibre post, composite core placement and thereafter, crown preparation (k) completed crown cementation (l) radiographic follow‐up at 3 years.

**FIGURE 9:**
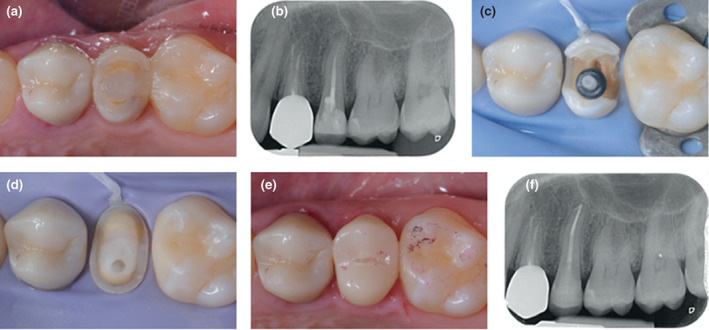
Lithium disilicate onlay (IPS e.max CAD) placement following root canal retreatment of 25 (a, b) preoperative occlusal view and long‐cone periapical radiograph (c) fibre post prior to cementation and core placement (d) completed core and onlay preparation with distal deep margin elevation (e, f) final CAD‐CAM restoration with postoperative occlusal view and follow‐up periapical radiograph.

Avoiding excessive post space preparation to maximize the preservation of dentine is a key principle in modern post‐endodontic restoration. Adapting the post to the existing parameters of the shaped root canal rather than creating a post space to accommodate a specific post is preferable, as extensive post space preparation affects the stability of the restored root filled tooth (Lang et al., 2006). In teeth with round root canals, the use of prefabricated, conically shaped and adhesively luted root canal posts, ideally without further post space preparation, is recommended (Figure 8). Up to now, there is no clinical evidence that the post material's rigidity affects the survival of root filled teeth or the occurrence of root fractures (Figueiredo et al., 2015; Martins et al., 2021). Outcome data for post‐retained restorations varies with respect to the amount of residual tooth structure, preparation of a ferrule design and final restoration (Bhuva et al., 2021), showing approximately 90% survival in the medium term (5–7 years) for teeth restored with fibre posts (Wang et al., 2019; Figure 9). However, one randomized clinical trial demonstrated a significant drop in the survival rate after 8 years leading to a cumulative survival probability of 58.7% for teeth restored with fibre posts and 74.2% for titanium posts after 11 years (Naumann et al., 2017).

Adaption of posts to the anatomy of unprepared, irregular shaped or flattened root canals can be achieved with customized and relined fibre posts or the use of fibre bundles. In the case of customized formed fibre posts, glass fibres are embedded in an interpenetrating polymer network (IPN) of PMMA and Bis‐GMA (everStick; Vallittu, 2009); such posts reveal higher fracture resistance than prefabricated solid fibre posts *in vitro* (Fokkinga et al., 2004). However, ageing affected bond strengths and surface nanohardness of the adhesive layer between composite and the polymerized fibre‐reinforced composite structure, indicating possible degradation effects depending on the monomer system used (Khan et al., 2018; Khan et al., 2019). Clinical data from teeth treated with such custom‐shaped posts are inconclusive. One study demonstrated lower success rates for customized fibre posts compared to solid fibre posts following 6 years of clinical service (Ferrari et al., 2012), whilst a further study showed similar 5‐year survival rates for cast gold posts and cores, solid fibre posts and customized fibre posts (Fokkinga et al., 2007).

The relining of fibre posts with resin composite modification using the root canal space has been suggested to facilitate the adaption of prefabricated posts in cases with nonuniform or greatly enlarged root canals (Grandini et al., 2003). Poor adaption of the post to the root canal parameters results in both a greater and less homogeneous thickness of the resin cement layer, increasing the risk of void inclusion, irregular contraction during polymerization and possible post dislodgement. Furthermore, a high resin cement thickness increases stress concentration inside the resin cement and decreased bond strength values *in vitro* (Dal Piva et al., 2017; Dal Piva et al., 2018), whilst relining of fibre posts results in increased bond strength in laboratory tests (de Souza et al., 2016; Farina et al., 2016; Macedo et al., 2010). However, data for the fracture resistance of root filled teeth restored with relined posts, remains inconclusive, even *in vitro* (Silva, Cabral, et al., 2021a; Silva, Versiani, et al., 2021b) and clinical data is still lacking. Furthermore, fibre post relining increases the clinical time needed for post‐endodontic restoration and creates another interface between the composite relining and root canal dentine.

Fibre bundles consist of clusters of 4–12 flexible prefabricated glass fibres, each with a diameter of 0.3 mm. This design allows the bundle to be inserted after the adhesive and composite have been applied, resulting in a flexible approach to evenly distribute the bundle across the canal, especially in the case of irregularly shaped anatomy. Previous studies demonstrated that bundled fibre posts exhibited intracanal adhesion as well as fracture resistance comparable to that measured for solid fibre posts (Bitter et al., 2019; Kul et al., 2020; Sturm et al., 2021) but with more homogeneous stress distribution (Yanik & Turker, 2022). Root filled maxillary central incisors with mesial and distal class III cavities restored with direct composite demonstrated comparable fracture resistance to those restored with solid and bundled glass fibre posts, and increased resistance compared to those restored without posts (Comba et al., 2021). In the case of weakened and flared root canals in immature teeth a combination of adhesively placed solid and bundled fibre posts contributed to fracture strength and stress distribution *in vitro* (Santos et al., 2022).

To date, no evidence exists that a specific post material or the rigidity of the post affects the outcome of post‐retained restorations. Posts mainly provide retention for the coronal restoration and are therefore indicated in teeth with extensive loss of coronal tooth structure. Substance preservation plays a key role in the survival probability of root filled teeth, and therefore, extensive post space preparation should be avoided, and the posts should be adapted to the shaped root canal space rather than the other way round. In cases of irregular‐shaped or extremely flared root canals adhesively placed customized fibre posts, as well as fibre bundles, can be used as an alternative option.

## ENDOCROWNS

Restorations that contribute to the structural integrity of root filled teeth, and preserve as much tooth structure as possible, improve long‐term prognosis. Preparation of a ‘ferrule design’ is of utmost importance to prevent tooth or root fracture of post‐endodontic crown restorations in severely compromised teeth (Juloski et al., 2012; Magne et al., 2017; Naumann et al., 2018). Ideally, this requires a minimum of 4–5 mm of supracrestal tooth tissue to provide a 2 mm ferrule preparation and secure a biological width of 2–3 mm, however, this is clinically not always available. Consequently, root filled teeth exhibiting significant tooth structure loss may require surgical crown lengthening or orthodontic extrusion. Surgical crown lengthening can critically alter the crown‐to‐root ratio, which contributes to stress and strain concentrations within the root dentine, and subsequently may negatively affect the fracture load behaviour and long‐term reliability of the post‐endodontic restoration (Avila et al., 2009; Gegauff, 2000; Tada et al., 2015). Therefore, endocrowns are a conservative approach for the restoration of root filled teeth without the need for post‐space or ferrule design preparation.

Recent systematic reviews and meta‐analyses demonstrate high success rates for endocrowns in molars (72–99%) and in premolars (68–100%) with a follow‐up range of 3–19 years, revealing no significant difference between tooth types (Thomas et al., 2020). Comparable survival and success rates for endocrowns and conventional post‐retained crown restorations were shown (Al‐Dabbagh, 2021), indicating that endocrowns are a reliable treatment option for compromised root filled molars and premolars. Recommendations for the preparation design include a depth of 3 mm for the central retention cavity with a divergence angle of 6–12° for more homogenous stress distribution (Abtahi et al., 2022; Tribst et al., 2021). The cervical margin width should be at least 2 mm, and prepared flat or slightly bevelled (Zheng et al., 2022).

Different materials have been used for the fabrication of endocrowns using CAD‐CAM technology. Lithium disilicate ceramic has been recommended frequently for this purpose, due to its favourable physical properties, good aesthetics and its predictable bonding to tooth tissue. However, hybrid ceramics or CAD‐CAM resin composites have a lower elastic modulus which is closer to that of dentine. Such hybrid materials could act as a stress absorber and reduce stress peaks within the root‐dentine and the restoration‐tooth interfaces, under clinical loads (Gresnigt et al., 2016; Rocca et al., 2016). A recent systematic review of *in vitro* studies demonstrated that CAD‐CAM resin composites had similar, increased, fracture resistance when compared to lithium disilicate ceramics, with less catastrophic failures also (Beji Vijayakumar et al., 2021). However, finite element analyses revealed stress concentrations proportional to the elastic modulus of the restoration material, with a higher stress concentration within the lithium disilicate ceramic, and less stress reaching the cement layer and residual tooth structure (He et al., 2021). Conversely, CAD‐CAM resin composites demonstrated a more uniform stress distribution but higher stress concentration inside the cement layer and surrounding tooth structure, which may lead to debonding (Yildirim et al., 2021). Fractographic analyses of clinically failed composite resin‐based endocrowns revealed a break‐up of the composite structure with reduced mechanical properties due to degradation processes and crack propagation leading to fracture events. These were mainly observed through the central occlusal groove, indicating less fatigue resistance of these restorations (Saratti et al., 2021). Due to the lack of long‐term controlled clinical studies, lithium disilicate ceramics are currently the material of choice for endocrowns, primarily due to its reliable bonding to the resin cement and its long‐term stability (El‐Ma'aita et al., 2022).

Endocrowns present a viable treatment option for root filled premolars and molars. These restorations permit the preservation of residual tooth structure, as post space preparation and placement, as well as preparation of a ferrule design, are avoided. Adequate adhesive luting, including proper isolation of the prepared tooth, are a prerequisite for this treatment alternative and its long‐term survival probability. Based on the currently available literature, lithium‐disilicate ceramics appear to be the material of choice for endocrown restorations.

## CONCLUSIONS

The evidence‐base for post‐endodontic restorative decision‐making remains complex and unclear. However, the interdependency of endodontic and restorative treatment is clearly established. Clinicians should consider both aspects of treatment equally, to give their patients the best outcomes. Moreover, engrained in the initial treatment planning process should be the final restorative plan for the tooth being treated.

As well as appreciating the importance of residual tooth structure, it is imperative to understand the relevance of tooth location and the number of proximal contacts on the survival of root filled teeth, and thereby, plan the post‐endodontic restoration appropriately. Therefore, it is also important to consider the prognosis of the teeth adjacent to that being treated, as their premature loss may influence its survival. It is fundamentally important for clinicians who undertake endodontic treatment to have a good understanding of how occlusal factors, and parafunctional stresses, may affect prognosis. The occlusion of each patient should be assessed prior to embarking on treatment, to identify and manage possible contributory factors, particularly in the case of cracked teeth.

The available evidence indicates that for posterior teeth, contemporary indirect techniques such as all ceramic crowns, onlays and endocrowns are as predictable as metal ceramic crowns. However, prospective studies with longer follow‐up periods are required to validate the performance of these restorations. In addition, particularly as dental materials evolve, the minimum required thickness of ceramics and composite materials, as well as cementation protocols require clarification for clinicians to provide the best outcomes.

The validity of minimal intervention and/or biomimetic endodontic‐restorative concepts and techniques has not been proven in clinical studies. However, this does not mean that these concepts should be dismissed. Indeed, surrogate measures from existing research suggest that there is a need for randomized clinical trials evaluating the long‐term survival of root filled teeth restored with these techniques. Clearly, how these are carried out, remains a challenge. The use of digital scanning offers an exciting opportunity for accurate volumetric assessment of residual tooth structure which could be utilized to offer tangible qualitative data for survival analysis which can then be conveyed to patients to facilitate decision making.

## AUTHOR CONTRIBUTIONS

All authors contributed to conceptualization, methodology, data curation, writing, review and editing.

## ETHICS STATEMENT

None.

## CONFLICT OF INTEREST

The authors have stated explicitly that there are no conflicts of interest in connection with this article.

## Data Availability

None
